# Enhancing Tool Wear Prediction Accuracy Using Walsh–Hadamard Transform, DCGAN and Dragonfly Algorithm-Based Feature Selection

**DOI:** 10.3390/s23083833

**Published:** 2023-04-08

**Authors:** Milind Shah, Himanshu Borade, Vedant Sanghavi, Anshuman Purohit, Vishal Wankhede, Vinay Vakharia

**Affiliations:** 1 Department of Mechanical Engineering, School of Technology, PDEU, Gandhinagar 382426, Gujarat, India; milind.smtmd20@sot.pdpu.ac.in (M.S.); vishal.wankhede@sot.pdpu.ac.in (V.W.); 2 Mechanical Engineering Department, Medi-Caps University, Indore 453331, Madhya Pradesh, India; himanshu.borade@medicaps.ac.in (H.B.); anshuman.purohit@medicaps.ac.in (A.P.); 3 Department of Mechanical and Aerospace Engineering, New York University, Brooklyn, New York, NY 11201, USA; vms8206@nyu.edu

**Keywords:** tool wear, generative adversarial network, Walsh–Hadamard transform, Dragonfly, Harris hawk, feature selection

## Abstract

Tool wear is an important concern in the manufacturing sector that leads to quality loss, lower productivity, and increased downtime. In recent years, there has been a rise in the popularity of implementing TCM systems using various signal processing methods and machine learning algorithms. In the present paper, the authors propose a TCM system that incorporates the Walsh–Hadamard transform for signal processing, DCGAN aims to circumvent the issue of the availability of limited experimental dataset, and the exploration of three machine learning models: support vector regression, gradient boosting regression, and recurrent neural network for tool wear prediction. The mean absolute error, mean square error and root mean square error are used to assess the prediction errors from three machine learning models. To identify these relevant features, three metaheuristic optimization feature selection algorithms, Dragonfly, Harris hawk, and Genetic algorithms, were explored, and prediction results were compared. The results show that the feature selected through Dragonfly algorithms exhibited the least MSE (0.03), RMSE (0.17), and MAE (0.14) with a recurrent neural network model. By identifying the tool wear patterns and predicting when maintenance is required, the proposed methodology could help manufacturing companies save money on repairs and replacements, as well as reduce overall production costs by minimizing downtime.

## 1. Introduction

The development of Industry 4.0, also known as the fourth industrial revolution, has significantly impacted the manufacturing sector in recent times. In the context of tool condition monitoring, Industry 4.0 has a significant role in improving monitoring systems’ accuracy and efficiency. One key aspect of Industry 4.0 in tool condition monitoring (TCM) is the use of advanced sensors and monitoring systems that are capable of collecting large amounts of data in real-time. These systems can be integrated with artificial intelligence (AI) and machine learning (ML) algorithms to analyze the data and provide insights into the condition of the tool [1]. For example, machine learning algorithms can be used to analyze the vibration patterns generated by the tool to identify any abnormal patterns that may indicate tool wear or breakage. Similarly, AI can be used to analyze temperature and pressure data to detect anomalies related to the tool’s condition [2]. This can result in significant improvements in efficiency and productivity, as well as reduce the risk of downtime due to unexpected tool failures or other issues. There are two main categories of tool condition monitoring techniques: direct and indirect. The measurement of physical characteristics that are directly connected to the tool or machine that is being monitored is involved in direct procedures. Indirect techniques of condition monitoring involve monitoring parameters that are indirectly related to the health of the machine or the component being monitored. These parameters can include temperature, pressure, voltage, current, and other physical or operational data that can provide insights into the machine’s condition. The indirect techniques of condition monitoring can also be combined with direct techniques to provide a more complete picture of the machine’s condition. For example, vibration analysis can be used in conjunction with temperature monitoring to detect abnormal vibration patterns that are related to temperature changes. Direct techniques are generally more accurate and reliable than indirect techniques, but they can be more complex and require specialized equipment to implement. On the other hand, indirect techniques are often more straightforward and easier to implement but may not provide as much detail about the tool or machine’s performance.

Vibration analysis is a powerful technique used in tool condition monitoring to detect any abnormalities or faults in a machine tool. By analyzing the vibration patterns generated by the tool, it is possible to identify various conditions such as tool wear, tool breakage, or poor tool performance [3,4]. Machine vision can monitor tool wear in real time using cameras and image processing algorithms. The authors [5,6] devised a method for detecting tool wear by analyzing the images of specimens manufactured through the machining process. Their approach identified surface roughness changes which could be further explored to estimate the remaining usable life of a tool. Moldovan et al. [7] provided an alternative technique. In the end milling process, they proposed a tool-flank-wear monitoring system that used the Euler number to distinguish between worn and unworn tool flanks under variable cutting periods. For a ball end milling operation, Zhang and Zhang [8] used an enhanced wear edge detecting method that achieved subpixel precision. Overall, the research indicates that machine vision is a viable technique for monitoring tool wear, with the potential to enhance the productivity and quality of industrial operations. However, there are obstacles to overcome, such as the inaccessibility of the cutting region during the machining process. In addition, certain direct TCM techniques need the cutting tool to be removed from the tool post, which may result in the misalignment of the tool during the subsequent operation [9]. There has been a plethora of attempts to establish a causal link between machining parameters and tool wear, and hence, support the use of indirect methods for monitoring tool condition [10]. In recent years, ML algorithms have grown in favor of a robust tool for predicting and monitoring the status of cutting tools. These algorithms have demonstrated significant results in a variety of aspects of tool condition monitoring (TCM), such as tool wear [11], surface quality [12], and material removal rate [13]. Real-time tool wears prediction using machine learning techniques enables the early identification and replacement of worn-out tools. These algorithms examine sensor inputs such as acoustic emission, vibration, and temperature to anticipate tool wear. The most often used methods for tool wear prediction are artificial neural networks (ANNs), support vector machines (SVMs), and random forest (RF) algorithms. ML algorithms may be used to evaluate the surface finish, which is a crucial quality parameter in machining operations. These algorithms then determine the surface finish based on process characteristics such as cutting speed, feed rate, and tool shape. The material removal rate is another parameter that determines the productivity of the machining process. ML algorithms can be used to enhance MRR by highlighting the required process parameters that yield the maximum MRR.

Studies have shown that machine learning algorithms can effectively predict tool wear rates in various machining operations, such as milling [14], turning [15], and grinding [16]. Hybrid models combining ML algorithms with optimization techniques have also been proposed and shown to have high accuracy in predictions [17]. SVM has also been used for TCM and has been shown to have good performance in detecting tool wear and predicting tool life [18]. Deep learning (DL) models have become a popular tool for TCM in recent years due to their ability to handle large amounts of data and to detect patterns and anomalies in data that are difficult to detect using traditional methods [19]. DL models such as convolutional neural networks (CNNs), recurrent neural networks (RNNs), and deep belief networks (DBNs) have been applied to TCM and trained on large datasets of machining processes to learn the patterns and relationships between tool conditions and tool wear. In addition, transfer learning techniques have been proposed for TCM, allowing for the transfer of knowledge from one machining process to another and improving the efficiency of the prediction process [20]. Despite these advantages, there are also limitations to the use of ML models for TCM. ML and DL models require large amounts of experimental data to be trained effectively. In the case of TCM, these data need to include detailed information about the machining process, tool conditions, and wear patterns. Recently generative adversarial networks (GAN) were developed by Goodfellow [21] and are capable of generating synthetic data that can supplement the real data used in training machine learning models. Compared to traditional techniques, GAN can be useful for tool condition monitoring in various ways. To begin, GANs can be used to generate synthetic data that can be used to supplement the real tool wear data used in training machine learning models. This is especially useful when there is a scarcity of real-world data for training. Secondly, GANs can be used to reduce the time and effort required for feature extraction while also improving prediction accuracy. Finally, with the availability of additional spectrograms (generated by GAN), deep learning models such as RNN can be developed, which was previously very tedious due to a lack of experimental data.

As per the available literature, the utility of GAN in the area of TCM has not been explored effectively. Therefore, to circumvent the issue of a limited dataset, the authors developed deep convolutional generative adversarial networks (DCGAN) to generate additional data on publicly accessible milling datasets from NASA’s Prognostics Centre of Excellence-Data Repository [22], which helped to train the ML models to predict the tool wear rate effectively. DCGAN is a type of GAN that uses convolutional neural networks (CNNs) to generate high-quality images. While GANs are a class of neural networks used for generating new data that resemble a given dataset, DCGAN specifically uses convolutional layers to improve the quality of generated images. The following is the author’s specific contributions:

1. To investigate the use of the Walsh–Hadamard transform and DCGAN for signal processing and spectrogram generation, respectively, and to assess their effectiveness in predicting tool wear.
2. To investigate and compare the performance of three different metaheuristic optimization feature selection algorithms through the selection of relevant features for tool wear prediction and evaluate their impact on prediction accuracy.
3. To compare the performance of three different machine learning models for tool wear prediction in conjunction with the Walsh–Hadamard transform, DCGAN, and the use of the selected features.
4. To develop a more accurate and efficient TCM system for tool wear prediction by integrating novel approaches using the Walsh–Hadamard transform and DCGAN, exploring metaheuristic optimization feature selection algorithms, and evaluating the performance of different machine learning models.

**Figure 1:**
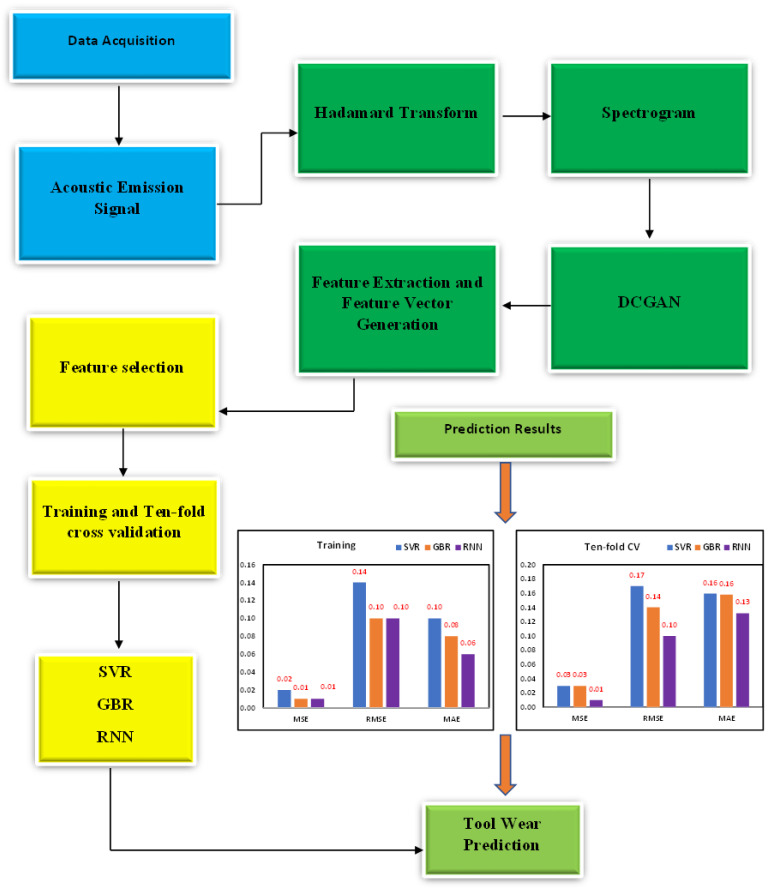
Proposed methodology.

The remaining sections of the article are structured as follows: Section 2 describes the methods and experiments, Section 3 analyzes the results, and Section 4 provides the conclusion. Figure 1 shows a flowchart of the proposed methodology.

## 2. Materials and Methods

### 2.1. Experimental Setup

The milling dataset for this study was obtained from the NASA Prognostics Center of Excellence-Data Repository [22]. This data set contains experiments conducted on a milling machine (Matsuura Machining Centre MC-510V) under different operating circumstances. Three distinct kinds of sensors were used to gather data: the AE sensor, the vibration sensor, and the current sensor. The dataset contains cutting parameters and tool wear measurements. The dataset is often used in metal cutting, tool wear prediction, and surface quality analysis-related research. Researchers utilize the data to construct and test machine learning models that can predict tool wear based on cutting parameters, optimizing machining processes and enhancing the efficiency of metal cutting operations. Typically, the NASA Milling dataset comprises measurements of cutting speed, feed rate, the depth of cut, tool geometry, and tool wear. Frequently, these data are preprocessed and divided into training and test sets, which are used to train and assess machine learning models. The table and spindle are equipped with AE and vibration sensors. All sensor signals are amplified and filtered prior to their transmission to the computer for data collection. The computer obtains the current sensor output from the spindle motor without additional processing.

The matrix of test parameters was determined by industry applicability and manufacturer-recommended values. Consequently, the cutting speed was set at 200 m/min, or 826 rpm per minute. Either 1.5 mm or 0.75 mm was chosen as the depth of the cut. In addition, two feeds of 0.5 mm/rev and 0.25 mm/rev were used, corresponding to 413 mm/min and 206.5 mm/min, respectively. Experiments on milling were performed using a 70 mm face mill and six KC710 inserts coated with TiC, TiC-N, and TiN for durability. The piece measuring 483 mm × 178 mm × 51 mm was composed of either cast iron or stainless steel. Except for a fresh set of inserts, all tests were performed under the same conditions but with a new set of inserts. This investigation focuses on the spindle’s AE signals when milling on cast iron. The experiment’s cutting parameters are shown in Table 1.

### 2.2. Acoustic Emission (AE) Signals

**Figure 2:**
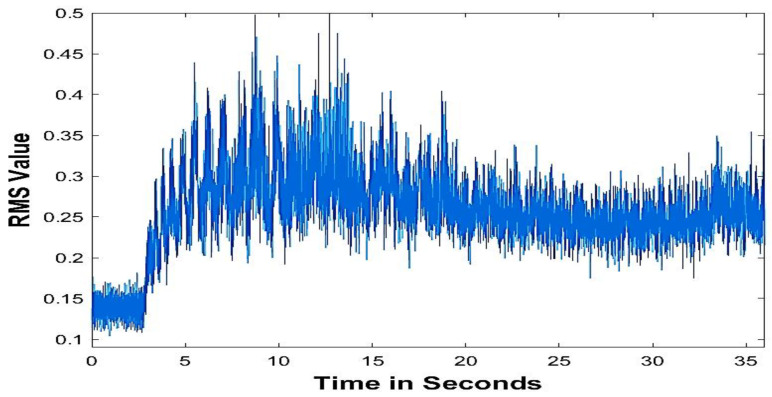
AE signal at depth of cut = 1.5 mm and feed = 0.25 mm/rev, work piece: cast iron.

Acoustic emissions refer to the spontaneous release of temporary elastic stress energy during the deformation of a material. This experiment collected AE data using an AE sensor with a frequency range of up to 2 MHz (model WD 925, Physical Acoustic Group). This sensor was clamped in place for support. A 50 kHz high-pass filter preamplifier (model 1801, Dunegan/Endevaco) was used to amplify the signal, which was subsequently amplified by a twin amplifier (model DE 302A). A custom-designed RMS meter sent the signal via cable to a high-speed data collection board (MIO-16). Figure 2 illustrates the recorded acoustic signals from the sensor.

### 2.3. Walsh–Hadamard Transform

Walsh–Hadamard transform (WHT) is a type of Fourier transform which decomposes any input vector into a superposition of Walsh functions [23]. In WHT, a linear transformation that transforms a sequence of numbers into a new sequence of numbers is mathematically performed to input signals. It is an orthogonal transformation, meaning that it preserves the inner product between the original and transformed sequences. Mathematically, the WHT can be represented by the following matrix equation:

(1) X=Hx

where *X* is the transformed sequence, *x* is the original sequence, and *H* is the Walsh–Hadamard transform matrix. The elements of the WHT matrix are given by:

(2) $$h(i,j)=\left(\frac{1}{\sqrt{n}}\right)\times {\left(-1\right)}^{i\&j},$$

where *i* and *j* are indices of the matrix, *n* is the length of the sequence, and & is the binary AND operation. The binary AND operation computes the bitwise AND of the binary representations of *i* and *j*.

The WHT is fast and efficient, as it can be computed in *O (n log n)* time using the Fast Walsh–Hadamard Transform (FWHT) algorithm. The WHT is used in various applications such as signal processing, fault diagnosis, cryptography, etc.

**Figure 3:**
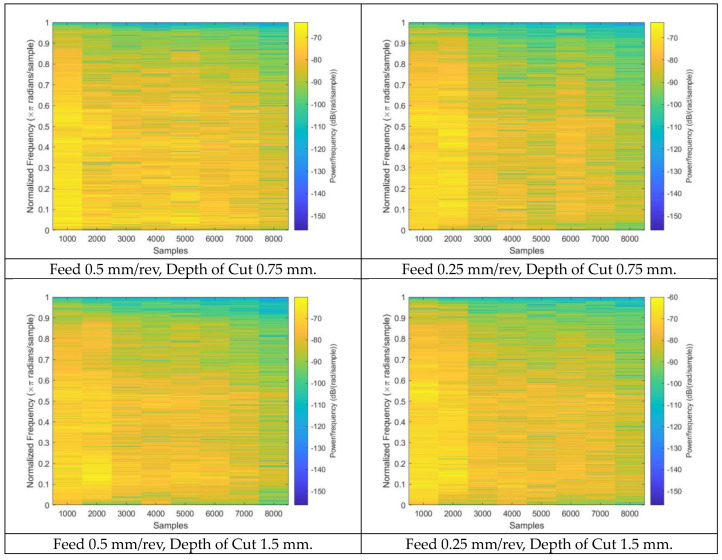
Scalogram generated from various milling operating conditions.

By analyzing the frequency and amplitude of the vibrations generated during the machining process, spectrograms can provide valuable insights into the condition of a tool. The color pattern in a spectrogram represents the intensity or amplitude of sound or vibration over time. On a spectrogram, darker or cooler colors represent lower amplitudes or quieter sounds or vibrations, whereas warmer or brighter colors represent higher amplitudes and louder sounds or vibrations. As a result, the color pattern in a spectrogram can show how the intensity of frequencies in sounds or vibrations changes over time. Darker colors near the bottom typically represent low-frequency components in the spectrogram. This is because low-frequency components have longer wavelengths and, thus, lower frequencies, which are lower on the spectrogram’s vertical axis. In contrast, high-frequency components are typically represented by brighter colors near the top of the spectrogram. This is because high-frequency components have shorter wavelengths and, thus, higher frequencies, which are higher on the spectrogram’s vertical axis. As a result, the position of the color intensity within the spectrogram can be used to identify a signal’s frequency content, with lower frequency components at the bottom and higher frequency components at the top. Figure 3 displays the spectrograms generated from measured AE signals with variations in their operating parameters.

### 2.4. Deep Convolutional Generative Adversarial Network (DCGAN)

When constructing an ML model for classification or regression, the availability of experimental data is a major concern. To circumvent this problem, the generative adversarial network (GAN) emerges as a potential tool. GAN is a deep learning technique developed for unsupervised learning in which the model learns to create new, previously unseen data samples that are comparable to the training data. It has a generator and discriminator network. The generator network generates data samples, while the discriminator network verifies them. The use of adversarial training between the generator and discriminator networks allows GANs to generate samples that are not only realistic but also diverse, capturing the variations of the target distribution [24,25]. There are several types of generative adversarial networks (GANs) that have been developed, each with a slightly different architecture or training procedure. The DCGAN is a specific type of GAN architecture that is designed for the generation of high-quality images. DCGANs use convolutional layers instead of fully connected layers, which allows them to learn spatial features in images. Instead of pooling layers and up-sampling layers, strided convolutions and transposed convolutions are used, which allows them to learn spatial features at multiple scales while avoiding a loss of information. DCGANs do not use fully connected layers in either the generator or discriminator networks, which reduces the number of parameters and helps to avoid overfitting [26]. The network comprises a generator and discriminator network. The DCGAN generator network converts a random noise vector, *z*, into an image, *G*(*z*). The noise vector is sampled from a Gaussian distribution and supplied to the generator to produce a new image. *D*(*x*) is a discriminator network that receives an input image, *x*, and produces a scalar value representing the likelihood that the input image is real. The generator aims to produce indistinguishable images from actual images, while the discriminator aims to differentiate between real and generated images reliably. The network uses convolutional and pooling layers, batch normalization layers, and activation functions to process the input data. The generator network uses a ReLU activation function in all layers except the output layer, which uses a tanh activation function to ensure that the output image pixels are in the range [−1, 1]. The discriminator network uses a Leaky ReLU activation function in all layers except the output layer, which uses a sigmoid activation function to produce a scalar output between 0 and 1. It uses a binary cross-entropy loss function to train the discriminator network, and the generator network is trained to maximize the loss function of the discriminator network to produce more realistic images [27]. The two networks are trained in an adversarial way, with the generator attempting to produce pictures that will deceive the discriminator and the discriminator attempting to distinguish the real and generated images properly.

**Figure 4:**
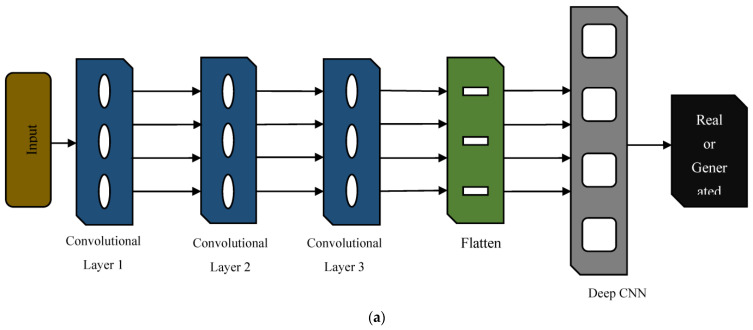

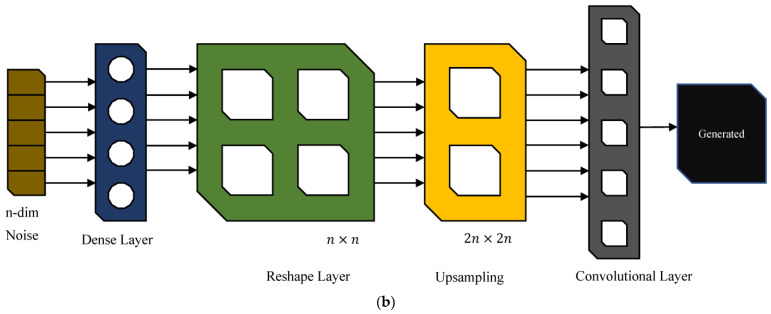
DCGAN Architecture [29]. (**a**) Discriminator, (**b**) Generator.

The mathematical formulation of the GAN can be expressed as:

(3) min G(z) max D(x) V(D,G)=E[logD(x)]+E[log(1−D(G(z)))]

where G(z) is the generator network, D(x) is the discriminator network, V(D,G) is the GAN objective, x is a real image from the training data, and *z* is a random noise vector. The first term, E[logD(x)], measures how well the discriminator can classify real images as real, while the second term, E[log (1−D(G(z)))], measures how well the generator can fool the discriminator. The generator and discriminator are trained alternately, with the generator trying to minimize the GAN objective and the discriminator trying to maximize it [28]. Figure 4a,b shows the architecture of the DCGAN model.

### 2.5. Machine Learning Models

Algorithms that are built to learn from data and make predictions or judgments based on that learning are known as machine learning (ML) models. Machine learning models can be broadly classified into supervised and unsupervised learning models. In supervised learning, models are trained on data with labels and known input and output attributes [30,31]. Decision trees, logistic regression, and Naïve Bayes are a few examples of supervised learning models. In unsupervised learning, models are trained on data that have no labels and just known input attributes. Unsupervised learning aims to find patterns or links in the data, for example, by grouping related data points. K-means and hierarchical clustering are two examples of unsupervised learning methods. In the present study, the authors explored the utility of the recurrent neural network (RNN), gradient-boosted regression (GBR), and support vector regression (SVR) for tool wear rate prediction.

#### 2.5.1. Recurrent Neural Networks (RNN)

A recurrent neural network (RNN) is a neural network that intends to handle the sequential input by retaining an internal state, or “memory,” to record relationships between sequence parts. In a conventional neural network, the input is systematically processed layer by layer to create an output. RNN, on the other hand, receives a succession of inputs and generates a sequence of outputs, with the network’s internal state changing at each step depending on the current input and the prior state. A typical RNN consists of an input layer, a hidden layer with a recurrent link, and an output layer. The recurrent connection allows information to flow from one time step to the next and is theoretically expressed as a function of the input, the prior state, and a set of learnable parameters. The structure of an RNN can be represented mathematically as follows [32]:

At time step *t*, the network takes as its input a vector *I_xt_* and an internal state vector *I_ht_,* and produces as its output a vector *I_yt_*:

$$I_{yt}=Ø(W_{yh}I_{ht}+W_{yx}I_{xt}+b_{y})$$

Here, Ø is the activation function, while $W_{yh}$, $W_{yh}$, and $b_{y}$ are learnt weight matrices and a bias vector, respectively, that translate the input and state to the output.

The internal state vector $I_{ht}$ is updated at each time step based on the input and the previous state, and can be represented as:

$$I_{ht}=Ӫ(W_{hh}I_{ht-1}+W_{hx}I_{xt}+b_{h})$$

$W_{hh}$ and $W_{hx}$ are learnt weight matrices, $b_{h}$ is a bias vector learned, and Ӫ is an activation function.

Each time step’s RNN output can be utilized as an input to the following time step, enabling the network to capture long-term dependencies in the sequence. Typically, the RNN’s final output was achieved by adding a fully connected layer or other post-processing to the output at the last time step.

#### 2.5.2. Gradient Boosted Regression (GBR)

Gradient-boosted regression is a machine learning algorithm that is used for predicting a continuous numerical value. It is a variant of the popular gradient boosting algorithm, which combines multiple weak models (also known as base learners or weak learners) to form a robust model that can make accurate predictions [33].

The algorithm works by fitting a weak model to the training data and then using the residual errors (difference between the predicted and actual values) to update the model. This process is repeated multiple times, with each subsequent weak model focusing on the residual errors of the previous model.

The final model is a combination of all the weak models, and is expressed as:

(4) $$F(x)=f_{1}(x)+f_{2}(x)+\ldots +f_{T}(x)$$

where *F*(*x*) is the final model, $f_{i}(x)$ is the *i*-th weak model, and *T* is the total number of weak models.

The objective function for the gradient boosted regression model is:

(5) $$L\left(y,F\left(x\right)\right)=\sum {\left(y-F\left(x\right)\right)}^{2}$$

where *y* is the actual value and *F*(*x*) is the predicted value.

The algorithm minimizes this objective function by using gradient descent, which involves taking the derivative of the objective function with respect to the model parameters ($f_{1},f_{2},\ldots ,f_{T}$) and updating the parameters to reduce the objective function.

Gradient-boosted regression has been shown to be effective in various applications, including predictive modeling, natural language processing, and image classification. It is a popular choice among machine learning practitioners due to its ability to handle large amounts of data and its ability to handle complex relationships between features.

#### 2.5.3. Support Vector Regression (SVR)

Support vector machines (SVMs) are supervised learning algorithms that are used for classification and regression tasks. They work by finding the hyperplane in a high-dimensional space that maximally separates the different classes [34]. The optimization problem for finding this hyperplane can be written as:

(6) maximize w,b,ξ

(7) $$\mathrm{subject}\;\mathrm{to}\;y_{i}(w\cdot x_{i}+b)\geq 1-\xi_{i}$$

(8) $$\xi_{i}\geq 0$$

where *w* and *b* are the parameters of the hyperplane, $x_{i}$ is a data point, $y_{i}$ is the label for that data point (either 1 or −1), and $\xi_{i}$ is the margin violation for that data point. The optimization problem seeks to find the hyperplane that maximally separates the classes while also allowing for some margin violations ($\xi_{i}$ > 0) to account for noise in the data.

The solution to this optimization problem can be found using the Lagrangian:

(9) $$L\left(w,b,\alpha \right)=\sum \alpha_{i}-\frac{1}{2}\sum \sum \alpha_{i}\alpha_{j}y_{i}(x_{i})\cdot y_{j}(x_{j})$$

where $\alpha_{i}$ is the Lagrange multiplier for the ith constraint. The solution to the optimization problem can then be found by taking the derivative of the Lagrangian with respect to *w* and *b* and setting it equal to 0.

SVMs have several benefits, including the ability to handle high-dimensional data and robustness to noise.

### 2.6. Metaheuristic Optimization Algorithms for Feature Selection

Feature selection is an important stage in many machine learning problems since it may enhance model performance by lowering the dimensionality of input data and removing noisy or unnecessary features. The objective of feature selection in machine learning is to discover a subset of features associated with a vast range of input data that could enhance the model’s performance. There are two main approaches to feature selection: feature ranking and feature selection using meta-heuristic optimization algorithms. The features are ranked according to their value or relevance to the target variable using feature ranking algorithms. Fisher score, information gain, and recursive feature elimination are well-known methods for ranking features. These approaches are straightforward and computationally efficient but do not always discover the optimal subset of characteristics for a specific situation. By contrast, feature selection using meta-heuristic optimization algorithms is a more advanced method that seeks the optimal subset of features by exploring a vast combinatorial space of feature subsets [35]. These algorithms include the genetic algorithm, the particle swarm optimization method, and the Dragonfly algorithm. The primary benefits of utilizing meta-heuristic optimization techniques for feature selection are that the algorithms are meant to examine the whole search space and identify the optimal subset of characteristics that maximize the objective function. In contrast, feature ranking algorithms may only find the most significant characteristics while ignoring the interactions between features, which might result in inferior solutions. Furthermore, meta-heuristic optimization techniques are resistant to noise and capable of handling high-dimensional feature spaces with many features. In contrast, ranking algorithms may not perform effectively in high-dimensional feature spaces or when a large number of irrelevant or duplicate characteristics are present [36]. The authors have conducted an exhaustive comparative study related to feature selection considering a description of algorithms; Dragonfly, Harris-hawk, and Genetic algorithms were used in this study as follows:

#### 2.6.1. Harris Hawk Optimization

Harris Hawk Optimization (HHO) is a metaheuristic optimization algorithm inspired by the behavior of Harris Hawks, a type of bird of prey known for their cooperative hunting techniques and this trait served as inspiration for the Harris Hawk Optimization (HHO) metaheuristic algorithm [37]. In order to solve a variety of optimization issues, including feature selection, the HHO method was developed. Feature selection is an approach used in machine learning and data mining to choose a subset of the most relevant features from a dataset to lower the dataset’s dimensionality. The objective is to identify the subset of features that results in the best performance of a classifier with respect to a selected performance measure, such as accuracy, precision, or recall. The HHO method searches for a high-dimensional binary space to discover the best feature subset. Each feature is represented as a binary value, 0 or 1, indicating whether it belongs to a feature in the feature subset. When searching across this space, the HHO algorithm employs a flock of birds, where each bird is meant to represent a possible feature subset. At each iteration, the birds are given an updated position that considers their present location, the locations of the other birds in the flock, and their optimum individual position. The Harris Hawk Optimization (HHO) method for feature selection has been mathematically formulated as follows:

Consider X as a data matrix that has m columns representing the features, *n* rows representing the samples, and *y* as the target vector that corresponds to *X*. One possible formulation of the optimization problem is as follows:

(10) minimizef(X)=g(X)+h(X)

where X is a binary vector representing the feature subset, g(X) is a function that assesses the performance of a classifier trained on the feature subset X, and h(X) is a function that penalizes the feature subsets with a high number of features. The function *g*(*X*) can be expressed as:

(11) g(X)=1−accuracy(X)

The function h(X) can be expressed as:

(12) $$h\left(X\right)=\alpha *{\left|\left|X\right|\right|}_{0}$$

where|| || is the L0-norm, which is the number of non-zero elements in X, and α is a positive constant that controls the trade-off between the accuracy and feature subset size. Each $X_{i}$ of the flock’s birds represents a possible feature subset. The following stages were carried out at each iteration of the algorithm:

Acceleration: Each bird $Z_{i}$ moves towards the best bird in the flock, $Z_{b}$, and also toward its own best position, $Z_{pi}$, in the following way:

(13) $$Z_{i}(new)=Z_{i}(old)+\gamma *(Z_{b}-Z_{i}(old))+\lambda *(Z_{pi}-Z_{i}(old))$$

where γ and λ are acceleration coefficients, and $Z_{b}$ and $Z_{pi}$ are the current best and personal best positions, respectively.

Velocity Update: The velocity of each bird is updated as follows:

(14) $$v_{i}(new)=Ø*v_{i}(old)+Z_{i}(new)-Z_{i}(old)$$

where Ø is the damping factor used to control the magnitude of the velocity update.

Position Update: The position of each bird is updated based on its velocity as follows:

(15) $$Z_{i}(new)=Z_{i}(old)+v_{i}(new)$$

Best Update: If the new position of a bird results in an improvement in the objective function, its personal best position is updated:

(16) $$iff(Z_{i}(new))<f(Z_{pi}),thenZ_{pi}=Z_{i}(new)$$

Steps (a–d) are repeated for a specified number of iterations or until a stopping criterion is met. The final result of the HHO algorithm is the best personal best position $Z_{pi}$ (new), which represents the best feature subset found during the optimization process.

#### 2.6.2. Dragonfly Optimization

The Dragonfly method (DA) is a metaheuristic optimization algorithm inspired by dragonfly flying patterns. The DA, developed in 2015 by Seyedali Mirjalili [38], is a novel optimization approach that considers ecological considerations. It can be used to find the best subset of features when building a model in machine learning problems where feature selection is needed.

**S.D.: -**

(17) $$D=\sum_{x\in X}\sum_{y\in Y}P\left(x,y\right)\frac{P(x,y)}{P\left(x\right)P\left(y\right)}$$

**Separation**

(18) $$S_{i}=-\sum_{j=1}^{N}P-P_{j}$$

where

*P* = Current Position

$P_{i}$ = Neighbouring $j^{th}$ position of *P*

*N* = Size of the Neighborhood

Alignment

(19) $$AV_{i}=\sum_{j=1}^{N}\frac{{V_{d}}_{j}}{N}$$

$V_{d_{j}}$ = Velocity of individual Neighborhood

*N* = Size of the Neighborhood

**Cohesion**

(20) $$Coh_{i}=\frac{\sum_{j=1}^{N}P_{j}}{N}-P$$

**Food attraction**

(21) $$F_{A_{i}}=P^{+}-P$$

$P^{+}$ = Position of food source

*P* = Current Position

**Food Distraction**

(22) $$F_{A_{i}}=P^{-}-P$$

$P^{-}$ = Position of enemy

The step Vector is modelled as:

(23) $$∆P_{i}^{t+1}=\left(S_{w}S_{i}^{t}+A_{v_{w}}A_{vi}^{t}+Coh_{w}Coh_{i}^{t}+F_{Ai}^{t}+eP_{i}^{t}\right)+I_{w}P_{i}^{t}$$

*t* = Iteration counter

$S_{w}$ = Separation Weight

$S_{i}^{t}$ = Separation of $i^{th}$ individual

$A_{v_{w}}$ = Alignment Weight

$A_{vi}^{t}$ = Alignment of $i^{th}$ individual

$Coh_{w}$ = Cohesion Weight

$Coh_{i}^{t}$ = Cohesion of $i^{th}$ individual

*F* = Food Factor

$F_{Ai}^{t}$ = Food Source of $i^{th}$ individual

*E* = Enemy Factor

$P_{i}^{t}$ = Position of enemy of $i^{th}$ individual

$I_{w}$ = Inertia Weight

Position of Dragonfly is updated as

(24) $$P_{t-1}=\left\{\begin{array}{c} -P_{t},r<T(∆P_{t+1})\\ P_{t},r\geq T(∆P_{t+1}) \end{array}\right\}$$

r = Random Number ∈ [0, 1]

(25) $$T\left(∆P_{t+1}\right)=\left|\frac{∆P}{\sqrt{\left(∆P^{2}+1\right)}}\right|$$

**Fitness Function**

(26) $$F=w_{1}\times Acc+\left(1-w_{1}\right)\times \left(\frac{F_{s}}{F-F_{s}}\right)$$

$w_{1}$ = Random parameter corresponding to accuracy weight

*Acc* = Accuracy

$F_{s}$ = Selected Feature

*F* = Feature Set

#### 2.6.3. Genetic Algorithm

The genetic algorithm (GA) is a search-based optimization technique inspired by genetics and natural selection [39]. In feature selection, a genetic algorithm (GA) may choose the subset of traits most relevant to a particular machine-learning task. Utilizing a GA for feature selection is useful because it can identify the optimal subset of features, resulting in improved performance and reduced computational cost. A GA-based feature selection procedure may be used to eliminate redundant or noisy features that could have a detrimental influence on the performance of a machine learning model. A further GA-based feature selection strategy may enhance the performance of a machine learning model by picking the most relevant features, leading to more accurate predictions. Depending on the specific implementation, the mathematical equations involved in a GA for feature selection may vary; however, the essential steps are outlined as follows:

- Initialization: Let *N* be the number of features and *X* denote the set of all features. Let *P* denote the population size and *p* denote the set of all feature subsets in the population. Each feature subset *p_i_* is represented as a binary vector, with the *i*th element presented as 1 if the feature is in the subset and 0 otherwise.
- Evaluation: *F*(*p_i_*) is the fitness function that estimates the performance of a machine learning model trained on the *i*th feature subset’s features. Accuracy measures, such as accuracy, F1-score, or AUC, can be utilized in the fitness function.
- Selection: Here, *p*′ denotes the set of chosen feature subsets, and p_selected denotes the collection of selected feature subset indices. The expression for the selecting stage can be written as:

(27) $$p_{selected}=select(p,F)$$

where the select function implements a selection operator, such as a roulette wheel selection or tournament selection.

d. Crossover: In this stage, $p_{crossover}$ represents the collection of feature subsets obtained by the crossover operation. The expression for the crossing step is:

(28) $$p_{crossover}=crossover(p^{\prime },F)$$

where the crossover function implements a crossover operator such as a uniform crossover or one-point crossover.

e. Mutation: Here, the expression for the mutation is written as:

(29) $$p_{mutation}=mutation(p_{crossover})$$

where the mutation function implements a mutation operator, such as flipping a random bit in the binary vector.

The evaluation, selection, crossover, and mutation processes are repeated until a stopping criterion is fulfilled. After a certain number of generations or when the population’s best-performing feature subset performs well, the procedure can be ended. The GA’s final result is the population’s best-performing feature subset.

## 3. Results

**Figure 5:**
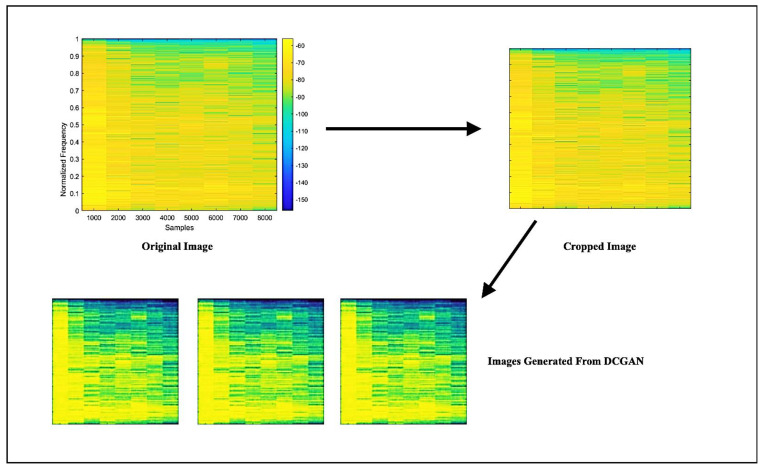
Synthetic scalograms generated from DCGAN.

In order to predict the tool wear of a specimen produced via the face milling process, 109 spectrograms were generated by employing the Walsh–Hadamard transforms on the AE signals. The spectrograms corresponding to distinct operating conditions are illustrated in Figure 3. Spectrograms are useful in predicting tool wear as they extract the meaningful and relevant properties of the signal generated during the machining process. The spectrogram displays the signal’s frequency content as a function of time, which allows the identification of specific patterns that correspond to tool wear. By analyzing the features extracted from the spectrograms, a machine-learning model can be trained to predict the level of tool wear for a given set of operating conditions. The features extracted through spectrograms are listed in Table 2 and were derived from the additional generated images obtained after applying DCGAN. From each original spectrogram, 100 images were generated, and the relevant features were extracted. A feature vector of size 10,900 × 7 was constructed, which can be used to train RNN, GBR, and SVR models to predict tool wear. The spectrogram generated after applying DCGAN is shown in Figure 5.

Table 3 and Table 4 exhibit sample feature vectors extracted from the generated spectrograms. As seen from both tables, the extracted features exhibit considerable variation with respect to various operating conditions performed on a milling machine. A standardized transformation of the feature vector was required to decrease bias and successfully train the models. During the standardized feature vector transformation process, the features were rescaled to ensure that the mean and the standard deviation would be equal to 0 and 1, respectively. Table 4 shows the sample feature vectors that were standardized. The Dragonfly, Harris hawk, and genetic optimization algorithms were used to identify the relevant features. These updated feature vectors were fed into SVR, GBR, and RNN models for flank wear prediction. To evaluate the tool wear prediction capabilities, three performance parameters, namely, the mean squared error (MSE), root mean squared error (RMSE), and mean absolute error (MAE), were computed.

(30) $$MSE=\frac{\sum {\left(A-P\right)}^{2}}{L}$$

(31) $$RMSE=\sqrt{MSE}$$

(32) $$MAE=\frac{\sum \left|A-P\right|}{L}$$

where *P* is the predicted value, *A* is the actual value and *L* is the number of observations.

Ten-fold cross-validation is a common technique that is used in machine learning to assess the performance of predictive models, such as those used for tool wear prediction. The utility of ten-fold cross-validation in tool wear prediction lies in its ability to provide a more accurate assessment of the model’s performance than simple holdout validation. Holdout validation involves splitting the data into a training set and a test set, with the model being trained on the former and tested on the latter. However, holdout validation can be sensitive to how the data are split, leading to variability in the performance estimates. Ten-fold cross-validation helps address this issue by repeatedly evaluating the model on different subsets of the data, thereby reducing the impact of the particular data split on the performance estimates. Table 5 displays the hyperparameter settings of the DCGAN and ML models used in the present study.

**Figure 6:**
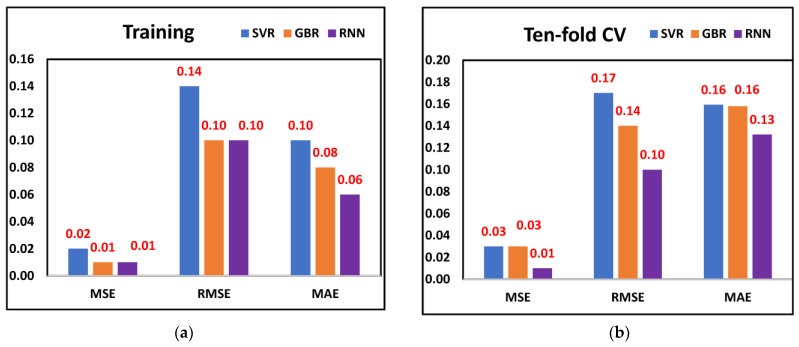
Prediction errors from Dragonfly selected features (**a**) Training (**b**) Ten-fold.

**Figure 7:**
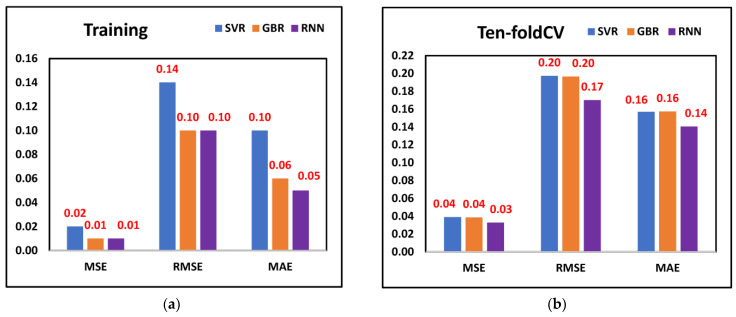
Prediction errors from Harris Hawk selected features (**a**) Training (**b**) Ten-fold.

**Figure 8:**
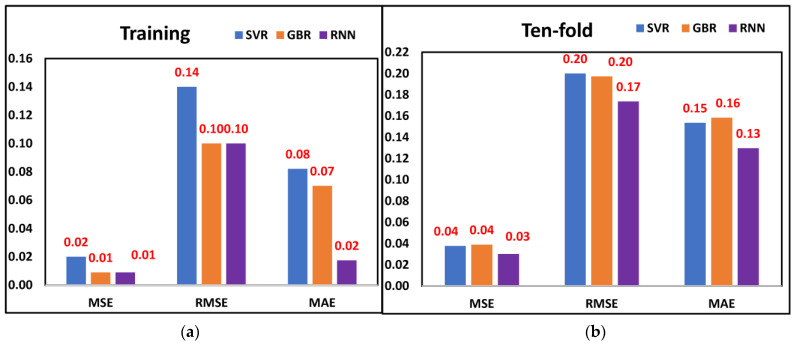
Prediction errors from genetic algorithm selected features (**a**) Training (**b**) Ten-fold.

The aim of this study was to investigate the efficacy of the proposed methodology for tool wear prediction. As discussed earlier, the features selected through metaheuristic optimization models were fed into three ML models. The MSE, RMSE, and MAE values are displayed in Figure 6a,b when training and ten-fold cross-validation was performed on all three ML models and considering the features selected through the Dragonfly algorithm. The least MSE (0.01), RMSE (0.10), and MAE (0.06) were observed from the RNN model, followed by GBR and SVR, respectively, when training was performed. A similar trend was observed when all three models were cross-validated. The least MSE (0.01), RMSE (0.10), and MAE (0.13) were observed from the RNN model. The results indicate that the RNN model was better for predicting tool wear since it provided significantly fewer prediction errors. Figure 7a,b displays the tool wear prediction errors when the features selected through Harris Hawk were fed into ML models. From the RNN model, the least MSE (0.01), RMSE (0.10), and MAE (0.05) were observed when training was performed, whereas when ten-fold cross-validation was considered, the least MSE (0.03), RMSE (0.17) and MAE (0.14) was observed from the RNN model. This finding suggests that the RNN model was better for predicting tool wear compared to SVR and GBR models when Harris Hawk features were considered. Finally, Figure 8a,b shows the tool wear prediction errors when features selected through the genetic algorithm were fed into ML models. The prediction results indicate that RNN was better compared to SVR and GBR as it provided the least MSE, RMSE, and MAE values after performing training and ten-fold cross-validation. By comparing the prediction errors of various machine learning models, the authors found that our proposed methodology integrating DCGAN, the Walsh–Hadamard Transform, and Dragonfly algorithm demonstrated reliable and promising results for tool wear prediction. Notably, RNN exhibited the lowest prediction errors when trained on the selected features from Dragonfly, Harris Hawk, and genetic algorithms, while the other two ML models also performed acceptably, whether trained on the selected features or using the ten-fold procedure. Overall, these findings suggest that our methodology can improve the accuracy of tool wear prediction and provides a valuable tool for industry practitioners.

The authors demonstrated that the performance of SVR, GBR, and RNN could identify prediction capabilities based on a proposed methodology. By evaluating the performance of multiple algorithms based on a proposed methodology, the authors were able to observe that deep learning, specifically RNN, was a viable approach for tool wear prediction as it produces the least prediction errors with all metaheuristic feature selection algorithms. To effectively demonstrate the utility of their proposed methodology, the authors prepared a comparison table (Table 5) that highlighted the significant differences and similarities among various research studies related to the same TCM dataset. This comparison table allowed readers to easily assess the performance of different machine learning algorithms for tool wear prediction and provided insights into the effectiveness of the proposed methodology. Overall, the authors’ approach thoroughly evaluated various machine learning algorithms and highlighted the potential of deep learning for tool wear prediction. There are certain reasons to choose SVR, GBR, and RNN as ML algorithms for tool wear prediction. The authors wanted to compare the performance of different machine learning algorithms for tool wear prediction. By applying multiple algorithms, the strengths and weaknesses of each algorithm were evaluated based on the proposed methodology. By comparing the performance of RNN with traditional machine learning algorithms, such as SVR and GBR, it was observed that deep learning could be a viable approach for tool wear prediction as it gives the least prediction errors with all metaheuristic feature selection algorithms. Further, a comparison table has been prepared (Table 6), which effectively demonstrates the utility of the methodology proposed by highlighting the significant differences and similarities among various research studies related to the same TCM dataset.

## 4. Conclusions

This investigation aimed to establish a precise method for predicting tool wear by analyzing the signals from AE sensors. To do so, the authors used Walsh–Hadamard Transform to remove noise from the measured signals before generating spectrograms from the filtered signals. However, obtaining sufficient training data was challenging due to limited experimental data availability. To address this issue, the DCGAN technique was used to generate additional spectrograms from the dataset. Following that, standard statistical features were extracted, and a feature vector was created. The authors utilized metaheuristic feature selection algorithms such as Dragonfly, Harris Hawk, and genetic algorithms to identify relevant statistical features. Finally, researchers assessed the effectiveness of our models with support vector regression (SVR), gradient-boosting regression (GBR), and recurrent neural network (RNN) models using training and ten-fold cross-validation techniques. The outcomes of this study are as follows:

(a) The RNN model with a Dragonfly algorithm feature has the lowest MSE (0.01), RMSE (0.10), and MAE (0.06) to predict tool wear when the training of ML models is carried out.
(b) When ten-fold cross-validation is performed, the tool wear rate prediction from the RNN model with features selected from the Dragonfly algorithm has the lowest MSE (0.01), RMSE (0.10), and MAE (0.13).
(c) Compared to the GBR and SVR models, the RNN model was found to have significantly fewer incorrect predictions regarding tool wear.
(d) The features chosen by the Dragonfly algorithm were superior to those selected by the Harris hawk algorithm and the genetic algorithm. This conclusion was reached after comparing the three algorithms.

The authors investigated three machine learning models, but other models may better predict tool wear. In the future, we need to explore different models, such as convolutional neural networks or transformer-based models, to improve the system’s accuracy. To enhance the performance of machine learning models, the authors could further optimize their hyperparameters. This could be accomplished through the use of techniques such as grid search or Bayesian optimization. The proposed TCM system could be improved by creating a real-time monitoring system capable of detecting tool wear in real-time and alerting operators to perform maintenance before significant damage occurs. In the manufacturing industry, this could lead to increased productivity and decreased downtime.

## Figures and Tables

**Table 1:** Face milling cutting parameters.

Parameters	Values
Depth of Cut	1.5 mm & 0.75 mm
Feed Rate	0.5 mm/rev & 0.25 mm/rev
Material Workpiece	Cast Iron & Stainless Steel J45

**Table 2:** Features extracted from the images.

Sr. No.	Feature
1	Mean Squared Error (MSE)
2	Root Mean Square Error (RMSE)
3	Pear signal to noise ratio (PSNR)
4	Visual Information Fidelity (VIF)
5	Mean Absolute Error (MAE)
6	Entropy (Ent)
7	Structural Similarity Index Measure (SSIM)

**Table 3:** Sample feature vector without standardization.

MSE	RMSE	PSNR	VIFP	MAE	Entropy	SSIM	Tool Wear
743.83	27.27	19.41	0.16	103.32	6.82	0.53	0.11
701.69	26.48	19.66	0.15	109.02	6.86	0.52	0.20
691.72	26.30	19.73	0.14	92.20	6.73	0.53	0.24
688.94	26.24	19.74	0.15	89.13	6.66	0.55	0.28
730.89	27.03	19.49	0.16	107.77	6.94	0.53	0.29
842.07	29.01	18.87	0.15	123.60	7.08	0.52	0.38
706.29	26.57	19.64	0.16	112.88	6.91	0.52	0.40
656.47	25.62	19.95	0.16	106.49	6.82	0.54	0.43
657.89	25.64	19.94	0.15	103.48	6.86	0.52	0.45
250.13	15.81	24.14	0.02	78.59	5.19	0.51	0.08

**Table 4:** Sample feature vector after standardization.

MSE	RMSE	PSNR	VIFP	MAE	Entropy	SSIM	Tool Wear
−0.10	−0.05	0.01	0.38	−0.31	−0.22	0.33	0.11
−0.39	−0.34	0.28	−0.35	0.06	−0.07	−0.26	0.20
−0.13	−0.08	0.03	−0.72	−1.22	−0.59	−0.13	0.24
−0.29	−0.24	0.18	−0.08	−1.35	−0.81	1.17	0.28
0.01	0.05	−0.09	0.39	−0.05	0.28	0.32	0.29
1.00	0.97	−0.91	−0.26	0.89	0.82	−0.99	0.38
−0.25	−0.20	0.15	0.21	0.34	0.18	−0.44	0.40
−0.58	−0.52	0.46	0.61	−0.04	−0.19	0.59	0.43
−0.55	−0.50	0.43	0.01	−0.34	−0.02	−0.28	0.45
−4.03	−4.84	5.77	−6.27	−1.98	−6.76	−1.41	0.08

**Table 5:** Hyperparameter settings of DCGAN and ML models.

Model	Hyperparameter	Value
DCGAN	Epochs	3000
	Batch Size	32
	Generator Dense Layer Activation Function	Rectified Linear Unit (ReLU)
	Generator Final Convolutional Layer Activation Function	Hyperbolic Tangent Function (Tanh)
	Discriminator Dense Layer Activation Function	Sigmoid Function
	Loss Function	Binary Cross Entropy
	Optimizer	Adaptive Moment Estimation (Adam)
RNN	Epochs	100
	Batch Size	32
	Optimizer	Adaptive Moment Estimation (Adam)
	Loss	Mean Squared Error (MSE)
	Learning Rate	0.0001
SVR	Kernal	Radial Bias Function
	Degree	3
	Regularization parameter	1
GBR	Loss	Squared Error
	Learning Rate	0.1
	No. of Estimators	100

**Table 6:** Comparative study with the published literature.

Reference	Sensors Used	Material of Workpiece	Algorithm	RMSE
Traini et al. [40]	All Sensors	Cast Iron	Logistic Regression	0.11
			Decision Forest	0.123
			Decision Jungle	0.116
			Boosted Decision Tree	0.122
			Neural Network	0.11
Yu et al. [41]	All Sensors	Cast Iron	Bidirectional LSTM	7.14
			BiLSTM-ED2	11.27
Hanachi et al. [42]	Current Sensor	Cast Iron	Sipos	0.42
			ANFIS	0.56
			RPF	0.22
Zhou and Sun [43]	Current Sensor	Cast Iron	LS-SVM	0.27
			KELM	0.14
			TAKELM	0.03
Proposed Work	Acoustic Emission	Cast Iron	RNN	0.1
			SVR	0.17
			GBR	0.14

## Data Availability

The authors used a publicly available milling dataset. Link: http://ti.arc.nasa.gov/project/prognostic-data-repository (accessed on 10 October 2022).

## Nomenclature

AE	Acoustic Emission
AI	Artificial Intelligence
ANN	Artificial Neural Network
CNN	Convolutional Neural Network
CV	Cross Validation
DA	Dragonfly Algorithm
DBN	Deep Belief Networks
DCGAN	Deep Convolutional Generative Adversarial Network
DL	Deep Learning
Ent	Entropy
GA	Genetic Algorithm
GAN	Generative Adversarial Network
GBR	Gradient Boosting Regressor
HHO	Harris Hawk Optimization
IoT	Internet of Things
MAE	Mean Absolute Error
ML	Machine Learning
MSE	Mean Square Error
NASA	National Aeronautics and Space Administration
PSNR	Pear signal to noise ratio
RF	Random Forest
RMSE	Root Mean Square Error
RNN	Recurrent Neural Network
SD	Standard Deviation
SSIM	Structural Similarity Index Measure
SVM	Support Vector Machines
SVR	Support Vector Regressor
TCM	Tool Condition Monitoring
VIF	Visual Information Fidelity
WHT	Walsh–Hadamard Transform

## References

[1] Zheng P., Wang H., Sang Z., Zhong R.Y., Liu Y., Liu C., Mubarok K., Yu S., Xu X. (2018). Smart Manufacturing Systems for Industry 4.0: Conceptual Framework, Scenarios, and Future Perspectives. Front. Mech. Eng..

[2] Naveen Venkatesh S., Arun Balaji P., Elangovan M., Annamalai K., Indira V., Sugumaran V., Mahamuni V.S. (2022). Transfer Learning-Based Condition Monitoring of Single Point Cutting Tool. Comput. Intell. Neurosci..

[3] Painuli S., Elangovan M., Sugumaran V. (2014). Tool Condition Monitoring Using K-Star Algorithm. Expert Syst. Appl..

[4] Jaen-Cuellar A.Y., Osornio-Ríos R.A., Trejo-Hernández M., Zamudio-Ramírez I., Díaz-Saldaña G., Pacheco-Guerrero J.P., Antonino-Daviu J.A. (2021). System for Tool-Wear Condition Monitoring in CNC Machines under Variations of Cutting Parameter Based on Fusion Stray Flux-Current Processing. Sensors.

[5] Vakharia V., Kiran M.B., Dave N.J., Kagathara U. Feature Extraction and Classification of Machined Component Texture Images Using Wavelet and Artificial Intelligence Techniques. Proceedings of the 8th International Conference on Mechanical and Aerospace Engineering (ICMAE).

[6] Patel D.R., Kiran M.B., Vakharia V. (2020). Modeling and Prediction of Surface Roughness Using Multiple Regressions: A Noncontact Approach. Eng. Rep..

[7] Moldovan O., Dzitac S., Moga I., Vesselenyi T., Dzitac I. (2017). Tool-Wear Analysis Using Image Processing of the Tool Flank. Symmetry.

[8] Zhang C., Zhang J. (2013). On-Line Tool Wear Measurement for Ball-End Milling Cutter Based on Machine Vision. Comput. Ind..

[9] Dutta S., Datta A., Chakladar N.D., Pal S.K., Mukhopadhyay S., Sen R. (2012). Detection of Tool Condition from the Turned Surface Images Using an Accurate Grey Level Co-Occurrence Technique. Precis. Eng..

[10] Jumare A.I., Abou-El-Hossein K., Goosen W.E., Cheng Y.-C., Abdulkadir L.N., Odedeyi P.B., Liman M.M. (2018). Prediction Model for Single-Point Diamond Tool-Tip Wear during Machining of Optical Grade Silicon. Int. J. Adv. Manuf. Technol..

[11] Umer U., Mian S.H., Mohammed M.K., Abidi M.H., Moiduddin K., Kishawy H. (2022). Tool Wear Prediction When Machining with Self-Propelled Rotary Tools. Materials.

[12] Svalina I., Simunovic G., Simunovic K. (2013). Machined Surface Roughness Prediction Using Adaptive Neurofuzzy Inference System. Appl. Artif. Intell..

[13] Nagaraj Y., Jagannatha N., Sathisha N., Niranjana S.J. (2020). Prediction of Material Removal Rate and Surface Roughness in Hot Air Assisted Hybrid Machining on Soda-Lime-Silica Glass Using Regression Analysis and Artificial Neural Network. Silicon.

[14] Zhang C., Yao X., Zhang J., Jin H. (2016). Tool Condition Monitoring and Remaining Useful Life Prognostic Based on a Wireless Sensor in Dry Milling Operations. Sensors.

[15] Dutta S., Pal S.K., Sen R. (2016). Tool Condition Monitoring in Turning by Applying Machine Vision. J. Manuf. Sci. Eng..

[16] González D., Alvarez J., Sánchez J.A., Godino L., Pombo I. (2022). Deep Learning-Based Feature Extraction of Acoustic Emission Signals for Monitoring Wear of Grinding Wheels. Sensors.

[17] Yang W.-A., Zhou W., Liao W., Guo Y. (2014). Prediction of Drill Flank Wear Using Ensemble of Co-Evolutionary Particle Swarm Optimization Based-Selective Neural Network Ensembles. J. Intell. Manuf..

[18] Wang Y., Zheng L., Wang Y. (2021). Event-Driven Tool Condition Monitoring Methodology Considering Tool Life Prediction Based on Industrial Internet. J. Manuf. Syst..

[19] Shah M., Vakharia V., Chaudhari R., Vora J., Pimenov D.Y., Giasin K. (2022). Tool Wear Prediction in Face Milling of Stainless Steel Using Singular Generative Adversarial Network and LSTM Deep Learning Models. Int. J. Adv. Manuf. Technol..

[20] Mamledesai H., Soriano M.A., Ahmad R. (2020). A Qualitative Tool Condition Monitoring Framework Using Convolution Neural Network and Transfer Learning. Appl. Sci..

[21] Goodfellow I., Pouget-Abadie J., Mirza M., Xu B., Warde-Farley D., Ozair S., Courville A., Bengio Y. (2014). Generative Adversarial Nets. In Proceedings of the International Conference on Neural Information Processing Systems (NIPS). arXiv.

[22] Agogino A., Goebel K. (2007). Milling Data Set.

[23] Beer T. (1981). Walsh Transforms. Am. J. Phys..

[24] Vakharia V., Vora J., Khanna S., Chaudhari R., Shah M., Pimenov D.Y., Giasin K., Prajapati P., Wojciechowski S. (2022). Experimental Investigations and Prediction of WEDMed Surface of Nitinol SMA Using SinGAN and DenseNet Deep Learning Model. J. Mater. Res. Technol..

[25] Creswell A., White T., Dumoulin V., Arulkumaran K., Sengupta B., Bharath A.A. (2018). Generative Adversarial Networks: An Overview. IEEE Signal Process. Mag..

[26] Alec R., Luke M., Soumith C. (2016). Unsupervised representation learning with deep convolutional generative adversarial networks. In Proceedings of the International Conference on Learning Representations. arXiv.

[27] Arora H., Jain S., Anand S., Rajpoot D.S. Augmentation of Images through DCGANs. Proceedings of the 2019 Twelfth International Conference on Contemporary Computing (IC3).

[28] Majtner T., Bajić B., Lindblad J., Sladoje N., Blanes-Vidal V., Nadimi E.S. (2019). On the Effectiveness of Generative Adversarial Networks as HEp-2 Image Augmentation Tool. Image Analysis.

[29] Chen L., Zhang J., Liang X., Li J., Zhuo L. Deep Spectral-Spatial Feature Extraction Based on DCGAN for Hyperspectral Image Retrieval. Proceedings of the 2017 IEEE 15th Intl. Conf. on Dependable, Autonomic and Secure Computing, 15th Intl. Conf. on Pervasive Intelligence and Computing, 3rd Intl. Conf. on Big Data Intelligence and Computing and Cyber Science and Technology Congress (DASC/PiCom/DataCom/CyberSciTech).

[30] Vakharia V., Castelli I.E., Bhavsar K., Solanki A. (2022). Bandgap Prediction of Metal Halide Perovskites Using Regression Machine Learning Models. Phys. Lett. A.

[31] Feijóo M.D.C., Zambrano Y., Vidal Y., Tutivén C. (2021). Unsupervised Damage Detection for Offshore Jacket Wind Turbine Foundations Based on an Autoencoder Neural Network. Sensors.

[32] Sudharsan R., Ganesh E.N. (2022). A Swish RNN Based Customer Churn Prediction for the Telecom Industry with a Novel Feature Selection Strategy. Connect. Sci..

[33] Friedman J.H. (2001). Greedy Function Approximation: A Gradient Boosting Machine. Ann. Stat..

[34] Cortes C., Vapnik V. (1995). Support-Vector Networks. Mach. Learn..

[35] Piri J., Mohapatra P., Dey R., Acharya B., Gerogiannis V.C., Kanavos A. (2023). Literature Review on Hybrid Evolutionary Approaches for Feature Selection. Algorithms.

[36] Kristiyanti D.A., Sitanggang I.S., Annisa A., Nurdiati S. (2023). Feature Selection Using New Version of V-Shaped Transfer Function for Salp Swarm Algorithm in Sentiment Analysis. Computation.

[37] (2019). Harris Hawks Optimization: Algorithm and Applications. Future Gener. Comput. Syst..

[38] Mirjalili S. (2015). Dragonfly Algorithm: A New Meta-Heuristic Optimization Technique for Solving Single-Objective, Discrete, and Multi-Objective Problems. Neural Comput. Appl..

[39] Mirjalili S. (2018). Genetic Algorithm. Studies in Computational Intelligence.

[40] Traini E., Bruno G., D’Antonio G., Lombardi F. (2019). Machine Learning Framework for Predictive Maintenance in Milling. IFAC-Pap..

[41] Yu W., Kim I.Y., Mechefske C. (2019). Remaining Useful Life Estimation Using a Bidirectional Recurrent Neural Network Based Autoencoder Scheme. Mech. Syst. Signal Process..

[42] Hanachi H., Yu W., Kim I.Y., Liu J., Mechefske C.K. (2018). Hybrid Data-Driven Physics-Based Model Fusion Framework for Tool Wear Prediction. Int. J. Adv. Manuf. Technol..

[43] Zhou Y., Sun W. (2020). Tool Wear Condition Monitoring in Milling Process Based on Current Sensors. IEEE Access.

